# Older persons’ experience with health care at two primary level clinics in Cape Town, South Africa: a qualitative assessment

**DOI:** 10.3399/bjgpopen20X101048

**Published:** 2020-07-01

**Authors:** Tsepo Motsohi, Mosedi Namane, Augustine Chidi Anele, Mumtaz Abbas, Sebastiana Zimba Kalula

**Affiliations:** 1 Division of Family Medicine and Palliative Care, School of Public Health and Family Medicine, Faculty of Health Sciences, University of Cape Town, Cape Town, South Africa; 2 Vanguard Community Health Centre, University of Cape Town, Cape Town, South Africa; 3 The Albertina and Walter Sisulu Institute of Ageing in Africa, Division of Geriatric Medicine, Department of Medicine, University of Cape Town, Cape Town, South Africa

**Keywords:** Aged, Primary health care, Qualitative research, South Africa, General practice, Quality of health care, Health services accessibility

## Abstract

**Background:**

Efficient methods of assessing older persons’ healthcare needs are required in busy public sector primary healthcare clinics in South Africa. These clinics are the main points of entry into the healthcare system. This study was part of a larger study to test the local applicability and adaptability of the World Health Organization's (WHO) Age Friendly Primary Care Toolkit for assessing and managing chronic diseases and common geriatric syndromes.

**Aim:**

To assess how older persons experience healthcare delivery at two primary healthcare clinics, and identify perceived gaps in health care to older people.

**Design & setting:**

A qualitative study at two primary healthcare sites in the suburbs of Cape Town, South Africa.

**Method:**

Focus group discussions (two at each facility) using an interview guide.

**Results:**

Analysed data were categorised into five themes: ‘despite the challenges, there is overall good care’; ‘communication gaps and the frustration of feeling unheard’; ‘the health service is experienced as being unreliable, stretched, and is difficult to access’; ‘there is a perception of pervasive structural ageism in the clinics’; and ‘there is a perception that the quality of care received is related to the profession of the healthcare provider’.

**Conclusion:**

Challenges of access and care for older clients at primary care clinics are linked to their age-specific holistic needs, which are not fully met by the current age-friendly arrangements. Measures should be taken at the clinics to complement the perceived good clinical care received, by improving access to care, making care appropriate to the need, reducing waiting times, and creating opportunities for older persons to feel respected and heard.

## How this fits in

Ageing is a global phenomenon associated with chronic comorbidity that requires chronic management. Primary health care is the entry point to health care for most of the population, and yet it is mainly geared towards managing acute health conditions. In South Africa, little is known about quality of care for older people in these facilities. This qualitative study gathered information from older persons on the quality of care received, and will help guide healthcare policy and clinical practice in South Africa and other low- to middle-income countries.

## Introduction

South Africa has the third highest proportion of persons aged ≥60 years in sub-Saharan Africa, numbering 4.4 million and representing 8% of the total population in 2015.^1^ As in other settings, there is a higher proportion of multiple chronic morbidities in this population, compared to the younger adult population, with hypertension, diabetes, and arthritis being the most common conditions.^1^ The care of patients with multimorbidity should be holistic, account for patterns of comorbidities, and emphasise continuity.^2,3^ This aim requires the development of efficient methods to assess patient care needs, and flexible care management support systems that respond to patients’ needs for different levels of support at different times.^4^ Major challenges in managing older patients in primary healthcare clinics include insufficient time and skills deficiencies. An investigation into primary care and care of older persons in Europe reported that, generally, healthcare systems in Europe are built on an acute episode model of care, which is ill-equipped to meet the fluctuating and complex chronic health problems of older people.^5^

The WHO has developed a scientifically sound toolkit to assist primary care centres to implement its age-friendly principles.^6^ The toolkit approaches assessment of the four geriatric giants – falls, immobility, incontinence, and memory impairment – and two major chronic diseases, namely hypertension and diabetes.^7^

Nurse-driven and doctor supported primary care clinics (community health centres [CHC]) are the entry point to health care for most of the South African population. Scant research has been conducted on the healthcare experience of older persons in South Africa. However, experience of overall healthcare responsiveness in the country has highlighted older persons’ dissatisfaction with access, communication, involvement in decision-making, and long waiting times.^8^ A recent study reported low levels of satisfaction and a lack of patient-centred health care, particularly in public healthcare facilities serving older people in low socioeconomic groups.^9^

This study is part of a larger multi-phase research project aimed at assessing the implementation of the WHO toolkit in two South African peri-urban primary care clinics. The primary objective of this phase of the project was to explore the experiences of older patients at the two clinics. The qualitative data gathered during this phase complemented a preceding exploration phase that collected quantitative data (study to be published). The very last phase of the project specifically assesses the applicability and adaptability of the toolkit. The current study was conducted before the toolkit implementation phase of the larger project. It used a predominantly inductive, Heideggerian phenomenological approach, wherein researchers did not bracket their biases and prior engagement with the question under study.^10,11^ This concept indicates that the meanings of the themes that the researcher induced were a blend of the meanings articulated by both participants' and researchers’ experiences of working in the two clinics.^10,11^ Thus, the researchers’ knowledge of the two clinics was explicitly used to contextualise and clarify the data. The approach is in contradistinction to a more traditionally detached, bracketed Husserlian phenomenological approach, wherein the researchers describe the phenomenon under study and bracket their biases.^10,11^ Bracketing involves the researchers holding their ideas, preconceptions, and personal knowledge in abeyance when listening to and reflecting on the experiences of participants.^10,11^

## Method

Patients aged >60 years who attended one of the two clinics were purposively recruited. The two facilities are the public sector CHCs of two suburbs in the Cape Metropolitan area: Hanover Park (HP), and Vanguard (VG) in Bonteheuwel. The total populations of HP and Bonteheuwel were 46 000 and 53 000, respectively, at the last country census (2011), with percentages of those aged ≥65 years being 6.8% and 6.4%, respectively.^12,13^ Both suburbs are low socioeconomic areas; 58% of the residents have a monthly household income of below ZAR3200 (US$221).^12,13^ Participants were recruited from clients (aged >60 years) whose clinical appointments were scheduled at the CHC just prior to, or on the day of, a focus group discussion. Written informed consent was obtained from each participant. The two focus group discussions conducted at each facility comprised eight to nine participants. The discussions were facilitated in quiet rooms on each clinic’s premises.

A dual moderator method was used to gather data. An experienced researcher conducted interviews in the languages spoken by the participants (isiXhosa, English, and Afrikaans) using an interview guide. Participants’ anonymity was maintained by using colours for names (for example, '*Red*') in the recordings and the transcripts. A second researcher (CA or MA) took fieldnotes. After each session, both moderators adjusted the interview guide to incorporate emerging new themes. The focus group discussions were audio-recorded and transcribed. Three of the researchers (MN, SK, and MA) listened to the audio recordings before all researchers reviewed the written transcriptions and made corrections. The interviews were conducted sequentially between August and November 2017, with the first two held at HP CHC and the other two at VG CHC. Saturation was reached when no new data topics were raised during the interviews. This occurred by the end of the second VG interview, which was the fourth overall interview. Confidentiality was further maintained through safe storing of the audio recordings and transcripts.

Three of the researchers (CA, MN, and TM) perused all four transcripts independently. Using a process of reading and rereading to immerse themselves in the data, each of the researchers generated codes for the data. The codes were amalgamated after consensus was reached through discussion. The amalgamated coding frame was used independently by TM and MN to re-read the data and assign preliminary themes. TM, SK, and MN then met to further refine the themes through discussion, debate, and critique.

## Results

A total of 33 participants were recruited to the study. Table 1 shows the demographic characteristics of the sample.

**Table 1.** Characteristics of study participants (*n* = 33)

**Age group, years**	***n* (%)**
<70	21 (63.6)
70–80	10 (31.3)
>80	2 (6.3)
**Facility**	**Median age, years (range)**
VG	66 (57–86)
HP	62 (60–79)
All	66 (57–86)
**Sex**	***n* (%)**
Female	25 (76)
Male	8 (24)
**Focus group**	**Number and sex of participants**
HP FG1	9 (3 females; 6 males)
HP FG2	8 (8 females; 0 males)
VG FG3	8 (8 females; 0 males)
VG FG4	8 (6 females; 2 males)

HP Hanover Park VG Vanguard FG Focus Group

The higher order themes that emerged from the data analysis are outlined below. These were: 1) ‘despite the challenges, there is overall good care’; 2) ‘communication gaps and frustration of feeling unheard’; 3) ‘the health service is experienced as unreliable, stretched, and difficult to access’; 4) ‘there is a perception of pervasive structural ageism in the clinics’; and 5) ‘there is a perception that the quality of care received is related to the profession of the health-provider’.

### 1. Despite the challenges, there is overall good care

A theme of experiencing good care was evident throughout the data. This assessment is based mainly on perceived respect:

*‘I was handled with respect, I do not want to name the doctor or the nurse by name, but they were very down to earth and friendly. I would recommend that the other people can come.’* (HP)

Notably, an assessment of good care was made, despite knowledge that patients have complained about the service and the cleanliness of the clinics:

*‘I can also say the same things as the others have said, because I first heard the people say the hospital is not good, but when I start coming here, it is a different thing* […] *so far, I have no complaints.’* (HP)

*‘For the past few years, I have been coming here, there were no problems for me, actually, and I received everything on time* […] *I am happy.’* (VG)

*‘For the number of people that have to be seen here* [at the health care facilities]*, the doctors and nurses are too few, it is difficult* […] *the government must employ more doctors and nurses.’* (VG)

### 2. Communication gaps and frustration of feeling unheard

This theme manifested in a variety of ways. First, it manifested at a level of having no voice about how the clinics’ systems could be improved:

*‘Cleaners are supposed to be here at seven o’clock, at least to start cleaning. They should not start cleaning while we are waiting* […] *We do not know what to say, we keep quiet.’* (VG)

*‘You must just keep your mouth shut, because you want it, you need the tablets, and there’s nothing else you can do’* (HP)

Second, frustration with feeling compelled to be silent was apparent at the clinical interface, which participants felt did not fully address their needs:

*‘For people to be treated with respect, pay more attention to people, to listen, and also to respect people when they tell you about their body, because you do not know my body, I know what I am feeling.’* (HP)

A disjuncture was apparent between the priorities of a doctor or clinical nurse practitioner and clients. Clients attach a measure of quality to being examined and having their specific complaints addressed meaningfully, through genuine acknowledgement complaints and adjustment of treatment:

*‘There is no examination, you are just given something without any examination. That is a problem to me. I always think about other people who might have the same tablets.’* (VG)

*‘My experience is when I say to the doctor that my feet pain and burns, then they say it’s arthritis, and they just give tablets* […] *And the next day, it is the same again (and I don’t know what it is) when I ask to be examined, they do examination with clothes on. That is all they do* […] *I am fed up.’* (HP)

Third, explicit and implicit reference was repeatedly made to the need for clients to be allowed to input into the clinics’ problemsolving and decision-making structures and processes:

*‘If the patient has not been served at the time when the chemist closed* [there are pharmacies in each clinic]*, returning the following day again, I think those people who were turned back must be helped first.’* (HP)

This desire to participate goes beyond internal facility processes and includes decision-making about where clinics should be located:

*‘So, both those communities attending here at Bonteheuwel, which is too small and too many patients. I think to make our lives better is to build us another hospital to serve our community from Langa from their side because it will be convenient for our elderly people. Our elderly people should walk all the way from the sports ground ‘til here, it is not on. We in Bonteheuwel can still take a taxi, but Langa people cannot take a taxi ‘til here. If the government can build another hospital for our people in Langa,* [Langa is a suburb in the Cape Metropole with a population of 52 401 (Census 2011)] *it will be a better service for our community as well.’* (VG)

The above statement, by participants in Langa, that the VG CHC belonged more to Bonteheuwel residents than them, was unique to the VG CHC data. There were no other notable differences in the data from the two clinics.

### 3. The health service is experienced as being unreliable, stretched, and difficult to access

A persistent view of the system as being stretched and unable to serve clients well was noted in the data, as well as nuanced understanding of reasons and challenges:

*‘For some or other reason, there wasn’t enough doctors, so they had to improvise and do other things, so at the end of the day, at four o’clock, we just finished with the* [sic]*, our examination with the doctors. They asked us to come the next morning for our medication. But it is sometimes, because of the amount of people coming here to the hospital.’* (HP)

*‘Like what the doctor told me one time, that if he would undress everybody, how much time would he have.’* (HP)

In a manner that overlaps with the frustrations of being unheard, clients reported having to resort to finding other means of tending to their needs and making the system work for them. This resourcefulness naturally caused problems when it bordered on corrupt practices:

*‘I want to talk about the cards* [appointment cards collected daily at reception and used to find patient files]*. That lady sits here, and she’s already early here, that man comes in and he does not come and sit here, and he gives the card to the one collecting cards, and he knows why he is taking that card because he knows there is something for him. The one that came afterwards, will be seen before the first one* [a few participants grumbling] [*…*] *that is, the people that must be seen to those who take the cards. What is right is right, what is wrong is wrong. A person who comes after me must be seen after me, even if he’s going to give R20* [a bribe]*.’* (HP)

*‘Sometimes you do not get the things you are supposed to get, you go to other places* [use of alternative service providers]*.’* (VG)

Difficulty in accessing the clinic was also expressed to be linked to the expensive public transport:

*‘You see the reason why the Langa taxis are charging the community so much? Because once they enter Bonteheuwel they will get in trouble, because here they work according to areas. One cannot work in another’s area. That is how taxis operate, otherwise there will be war. There was a gentleman that offered* [a] *lift for Langa, to bring them here and take them back for R10, and the people did not want to take that opportunity.’* (VG)

### 4. A perception of pervasive structural ageism at the clinics

Explicit and implicit references to ageism are made throughout the data. A view was consistently expressed of being treated differently because of being older. This differentiation was most explicitly noted in how staff members communicate with older clients:

*‘For an older person, you’ll find out that they* [staff members] *are abrupt’*. (HP)

*‘Like, if people ask a question, you are told to just get out. Plain, simple question, and they will chase you out, and sometimes it’s grown adults and they are youngsters, disrespect in other words, you know.’* (HP)

Previously mentioned unmet client expectations during consultations can also be viewed through an ageist lens. In the following instance, a patient’s physical complaints are dismissed as being due to old age:

*‘I take it as, I am old, they can’t help me, so I can only get the high blood tablets, pain tablets* […] *You see, that’s what they told me* […] *it’s all about age. Everything is just to do with age. I think I know my own body and age is in your mind, also. Sometimes I feel good and I feel young; when I feel sick, I feel down, you see. So, I do not think they cannot help, if they want to help you, they can.’* (HP)

Nonetheless, as noted below, the participants acknowledge some efforts made by the facility to process and prioritise older patients, and the challenges experienced when attempting to do so:

*‘There is a concern regarding wheelchair patients, which I think those people must get first privilege. Even if it is at reception to be helped first, at the doctor to be seen first or to be seen at the pharmacy. Yes, at the pharmacy, they do get the privilege, but at the doctor and at reception they do not get it. We, as elderly, can still walk and do whatever we want to, but those poor people sitting on* [a] *wheelchair, do not have the chance to lift themselves up, ever* […] *If only they could look at that type of people. I know we’re busy with elderly people, but I also request that we look at that type of people*.’ (VG)

However, what was felt to be missing from the facility's efforts is a lack of consideration of the holistic needs of older people and the contextual challenges they face, which affects access to care and needs attention:

*‘The old people can’t sit there all time. They have to go to the toilet. Now they come back, and the name was called already, now they have to wait again. I saw it, they put the folder on one side. Now the old people have to wait until they decide to call their names again. When they go to the window, I tell them your name has been called … They don’t ask, where have you been or even help them* […] *’* (HP)

Being a carer for grandchildren poses challenges when older people must access services that keep them away from home for extended periods of time. The subtext is that this should be considered when making decisions about patient processing in the clinics:

*‘They can see how people sit and wait, and many a times people do not have something to eat. And you can see people are frustrated, they have children with them, they need to fetch kids from school.’* (HP)

### 5. There is a perception that the quality of care received is related to the profession of the healthcare provider

This theme manifested most pointedly in the level of importance clients ascribed to doctors, compared to other healthcare staff categories:

*‘There are no complaints, you can’t say anything wrong about the doctors. They look very good after us. If it wasn’t for them, then where would we have been?’* (HP)

This importance is framed within a narrative of quality of clinical care.

*‘I had to wait for four hours, and* [was] *helped on the fifth hour, and I saw a sister* [professional nurse]. *That was not nice, because we cannot talk everything to them. You want to see the doctor and talk to them, and your mind is set on the doctor. When you talk to the sister, they will always say to you,* *“Oh, that is nothing, it will go away* *”.* *'* (HP)

*‘Sister with the red things?* [epaulets] *No. We like to be seen by doctors, because they thoroughly examine you.’* (VG)

Interestingly, this perception and differential valuing loses sight of the quality of care and services that can be provided by competent nurse practitioners during clinical consultations. However, there are contradictory observations in the dataset, where there is an expressed satisfaction with the treatment given by nurse practitioners:

*‘I am very glad for the treatment from all the nurses.’* (HP)

Nonetheless, there is a tendency throughout the dataset of perceiving and experiencing certain staff categories as less helpful, and having worse attitudes than others.

## Discussion

### Summary

This study has shown that overall, older clients experience satisfactory health care at the two primary care clinics. Despite policies of inclusivity and distributive leadership being in place for public health facilities, the participants noted persistent gaps in communication about services and involvement of older clients in decision-making processes. Challenges of access and processing that the participants experienced seem to pertain to circumstances and needs that are specific to older patients. Some challenges were related to a perceived ageism, despite both clinics having age-friendly policies in place. A perception that doctors give better quality of service than other categories of staff, despite explicit acknowledgement of good service from these other categories, was unsurprising because of the high regard patients have for doctors’ qualifications compared to those of other healthcare providers.^14^

### Strengths and limitations

This is the second qualitative study in South Africa of older clients’ experience and satisfaction with health care provided at primary care services.^9^ The use of a fluent, multilingual focus group moderator was a notable strength, however, there were some limitations to the study. No computerised coding program was used, which may have affected the rigour of the coding. However, this omission was mitigated by the involvement of the three researchers in the coding, and a consensus on codes that was reached. Saturation of the data was achieved during collection. In addition, the previously mentioned interpretivist, Heideggerian phenomenological approach offers a useful framework for other researchers working on the topic in similar settings.^10,11^

Intrinsically, the purposive sampling technique used for the qualitative study will have led to some selection bias. The recruitment was done around the dates set for the interview, which means that patients who were not present on those days were not afforded the opportunity to participate. Furthermore, the low representation of participants from other suburban areas served by the two clinics meant that their views and experience were not captured. The generalisability of the findings is limited by the intrinsic nature of the qualitative study.

### Comparison with existing literature

Several themes emerged from the data that were congruent with the researchers’ experience of the service in the two clinics. Some findings also resonated with those in the literature on the experience of older clients in health care. Communication gaps are commonly a root cause of a number of organisational challenges, particularly gaps pertaining to inclusive dialogue with clients and stakeholders.^15^ Both clinics have well established avenues for communication with patients, including regular meetings with patient representative health committees, open door policies of leadership, and well-signposted 'compliments and complaints' procedures that are health policy requirements.^16^ Nonetheless, the data suggested that communication is stifled, rather than facilitated, at the interface with individual service points, such as consultations and encounters with reception.

Explicit in the VG participants’ comments were references to what they felt was a 'non-inclusive' planning and decision-making processes that resulted in the facility being located where it is. However, one of the researchers (MN) is familiar with the well-documented history of a series of broad, multiple stakeholder engagements that took place during the planning stages of the construction of VG CHC. Therefore, it is conceivable that participants who expressed such views may perceive that they have been marginalised, despite these inclusive engagements.

Regarding structural ageism, there is well-documented evidence in the literature of clinicians’ views of older clients as having complex healthcare needs and being difficult to treat.^17^ This may explain some of the observed attitudes and behaviours of healthcare providers.^17^ Nonetheless, the theme of structural ageism should be juxtaposed with contrary and equally thematic perceptions of good care. The ‘good’ care expressed was largely a show of sympathy for healthcare professionals who render services in overcrowded facilities with limited resources. The picture is, therefore, one of complexity due to this combination of positive and negative perceptions held by the participants.

Resonance of the findings with other studies is encouraging.^18^ This includes client perceptions of doctors’ superiority over other healthcare workers.^14^ Elsewhere, patients have been found to associate better interpersonal skills with nurse practitioners and physician assistants, while associating better technical skills with doctors.^14^ In this study, the perceptions of poor interpersonal skills were attributed to nurses more than doctors. Interestingly, the clients’ trust of primary care-based physician associates in the UK were more positive, and this seems to be related to an overall trust of the NHS to employ healthcare workers who will provide quality care.^19^ Lastly, Kelly *et al* conducted a study in similar socioeconomic areas of Cape Town, in which comparable themes emerged.^9^ These themes included ageist staff attitudes, long waiting times, and hurried consultations with no examinations.^9^

### Implications for practice and research

This qualitative study was part of a large study, which assessed the feasibility of introducing the WHO age-friendly primary healthcare centres toolkit to a South African primary care setting. The study's outcomes suggested that it would be reasonable to employ this research model to assess clients’ experiences after implementation of the toolkit as planned. The results also suggest helpful focus areas to be included in existing client satisfaction surveys of primary care services. Both clinics in the study prioritise accelerated access for older clients, children, and clients living with disability. However, the findings on access suggest that the implementation of these policies has deficiencies, and that other contextual and age-specific needs of older patients — such as being carers for grandchildren when the parents are working (limiting the waiting period at a facility) — may need consideration when planning for future services.

Other innovative methods of communication of services and access (such as digital, community radio, recorded audio, and volunteer ushers) may be helpful for addressing communication gaps that were highlighted in the study. Finally, to address ageism, the implementation of values clarification workshops for healthcare and frontline workers may be indicated. Such implementation could, in turn, yield useful research questions about its effectiveness.
